# A Relation Extraction Framework for Biomedical Text Using Hybrid Feature Set

**DOI:** 10.1155/2015/910423

**Published:** 2015-08-10

**Authors:** Abdul Wahab Muzaffar, Farooque Azam, Usman Qamar

**Affiliations:** National University of Sciences and Technology (NUST), H-12, Islamabad 44000, Pakistan

## Abstract

The information extraction from unstructured text segments is a complex task. Although manual information extraction often produces the best results, it is harder to manage biomedical data extraction manually because of the exponential increase in data size. Thus, there is a need for automatic tools and techniques for information extraction in biomedical text mining. Relation extraction is a significant area under biomedical information extraction that has gained much importance in the last two decades. A lot of work has been done on biomedical relation extraction focusing on rule-based and machine learning techniques. In the last decade, the focus has changed to hybrid approaches showing better results. This research presents a hybrid feature set for classification of relations between biomedical entities. The main contribution of this research is done in the semantic feature set where verb phrases are ranked using Unified Medical Language System (UMLS) and a ranking algorithm. Support Vector Machine and Naïve Bayes, the two effective machine learning techniques, are used to classify these relations. Our approach has been validated on the standard biomedical text corpus obtained from MEDLINE 2001. Conclusively, it can be articulated that our framework outperforms all state-of-the-art approaches used for relation extraction on the same corpus.

## 1. Introduction

With the massive information and knowledge hidden in the biomedical field, in the form of publications, that is growing exponentially, it is not possible for researchers and practitioners to keep themselves updated with all the developments in any specific field [1, 2]. The emphasis of biomedical research is shifting from individual entities to whole systems, with the demand of extracting relationships between entities, for example, protein-protein interaction, diseases genes from biomedical text to generate knowledge [3, 4]. Manual effort to transform unstructured text into structured is a laborious process [5]. Automatic techniques for relation extraction provide a solution to the problem [6].

A number of relation extraction techniques for biomedical text have been proposed [7–10]. These techniques are broadly categorized into four groups, that is, cooccurrence based, pattern-based, rule-based, and machine learning based approaches.

The simplest approach to identify/extract relations between entities is cooccurrence that identifies cooccurring entities in a sentence, abstract, or document [11]. Pattern based systems rely on a set of patterns to extract relations; these patterns can be defined manually as well as automatically. Manual patterns are defined by domain experts, which are a time-consuming process and have low recall [12]. To increase the recall of manually generated patterns, automatic pattern generation is used. Automatic pattern generation can use bootstrapping [13] or generate directly from corpora [14]. In rule-based systems, a set of rules can be built to extract relations [15, 16]. Rule-based systems can be defined in both ways, that is, manually and automatically. When the annotated corpora on biomedical is available, machine learning based approaches become more effective and ubiquitous [17, 18]. Most approaches use supervised learning, in which relation extraction tasks are modeled as classification problems. Broadly, any relation extraction system consists of three generalized modules that is, text preprocessing, parsing, and relation extraction.

This paper presents a detailed feature set to extract the relations between disease and treatment from biomedical text. This representation model is a hybrid as it uses the bag of word, natural language processing, and semantic representation to extract biomedical relations. Our framework is validated on the standard corpus form [19]. By presenting the implementation for three relations in the corpus that is, cure, prevent, and side effect relations.

## 2. Literature Review

Generally, the relation classification in biomedical domain is done by three methods, that is, supervised, semisupervised, and unsupervised. Major work in biomedical domain is done on protein-protein interaction, or protein and gene relation, while not a lot of work has been cited in disease-treatment relations.

Huang et al. [20], the authors, proposed a new hybrid approach to extract protein-protein (P2P) relations from biomedical scientific papers. This approach is a combination of shallow parsing and pattern matching. Based on shallow parsing analysis, using syntactic and semantic constraints, appositive and coordinative structures are interpreted. Long and complex sentences are then divided into smaller ones. In the end, the greedy pattern matching algorithm has been used to extract relations from shorter sentences, and patterns are generated automatically. This technique achieved an average *F*-score of 80% on individual verbs and 66% on all verbs. As stated by the use of shallow parsing analysis, remarkable improvement in pattern matching is noticed. Author mentioned 7% improvement of both *F*-score and precision of their approach compared to traditional pattern matching algorithms and achieved a performance which is comparable to the best of these systems. This approach was complex and designed for a small domain of protein-protein interaction, as patterns were developed to extract proteins only.

Frunza and Inkpen [21] used the integration of biomedical and medical knowledge for the discovery of semantic relations, from biomedical sentences, which occurs between diseases and treatments. Cure, prevent, and side effect relations are the semantic relations considered to be extracted between entities (disease, treatment). The authors claimed better results compared to the previous studies done on this topic. Results showed different figures for each of the three relations mentioned: accuracy for cure relation is 95%, prevent relation has 75% accuracy, and 46% accuracy for side effect relation has been claimed. The approach lacks detailed analysis as it does not give the *F*-score.

Sharma et al. [22] primarily focused on the task of identification and extraction of relations between entities present in biomedical literature. The paper proposed a verb-centric algorithm, unlike cooccurrence patterns or manual syntactic rules, as done in previous biomedical relation extraction work. No rule-based approaches are required, as algorithm identifies the main verbs in the sentences. The entities involved in relations are then identified using a dependency parse tree with syntactic and linguistic features. As claimed by the authors, this technique can extract the relations from complex sentence structures effectively. The algorithm is evaluated on multiple biomedical datasets prepared using MEDLINE, and the average *F*-score achieved is almost 90%. The main problem with this approach was that it caters only to words from the part of speech. No preprocessing was done, that is, stop word removal, or stemming, and so forth was done. Also, no standard dataset was used to evaluate the technique.

Ben Abacha and Zweigenbaum [23] explained the extraction of semantic relations from medical text. The scope of relation extraction is only between disease and treatment entities. The authors propose an approach, which is a hybrid in nature; that is, it employs two different techniques to extract the semantic relations. In the first technique, relations are extracted by patterns based on human expertise whereas, in the second one, relations are extracted by a machine learning technique based on Support Vector Machine classification. This new hybrid approach mainly relies on manual patterns when available relation examples are less, while feature values are used more when the number of available relations examples is sufficient. The authors claimed an overall *F*-measure of 94.07% for cure, prevent, and side effect relation extraction. Due to pattern based approach, the domain specific results are generated. Also, since the feature extraction was based on dataset, it may not perform well on disease-treatment relation for some other datasets.

Ben Abacha and Zweigenbaum [24] present a platform, MeTAE, for identification of medical entities and medical relations linking those entities. The approach is based on linguistic patterns and domain knowledge. The proposed approach contains two main parts. First part deals with the medical entities' recognition and in the second part exact semantic relations between any two identified medical entities are extracted. The identification of medical entities is achieved by an extended use of MetaMap. The results with the simple use of MetaMap and extended use of MetaMap are compared and the latter improved the precision by 19.59%. The extraction of medical relations is based on linguistic patterns which are constructed semiautomatically from a corpus chosen using semantic criteria. 16 types of medical entities are identified to evaluate the ability of the proposed system. In order to assess the system, the extraction of treatment relations between a medication and a disease is also taken into account. The results claimed by this research are encouraging as compared to similar research works in the literature with a precision of 75.72% and a recall of 60.64%. The problem with this approach is that named entity recognition phase does not extract all the named entities for which relations are to be extracted, so it further decreases the precision and recall of the approach.

Yang et al. [25] proposed a system with four main modules. The first module deals with the named entity recognition; here authors extract five entity types which are foods, chemicals, diseases, proteins, and genes. The second one is the relation extraction module to extract binary relationships between the entities; this is based on the verb-centric approach. The third module focuses on the polarity and strength analysis of relationships. In order to capture the syntactic, semantic, and structural aspects of relations, unique features have been constructed by authors. Support Vector Machine and support vector regression are used in this step. A user interface has been developed to integrate and visualize extracted relationships to intuitively observe and explore the previously extracted relations. Evaluation of the first three modules of this system exhibits its efficiency. The named entity recognition has an *F*-score of 89% with equal precision and recall, relationship extraction task has an *F*-score of 90.5%, and the accuracy of relationship polarity was 91%, while 96% strength level was rated in relationships. This technique lacks as it did not use a standard dataset, rather they built their own dataset and also no proof of results was stated in the paper.

Kadir and Bokharaeian [26] proposed a new technique to extract relations between biological and medical entities from biomedical documents. This technique is hybrid in nature, combines different relation extraction approaches, and extracts simple as well as complex relations between the pair of entities. The proposed hybrid approach combines rule-based, kernel based, and cooccurrence based methods. Combiner and classifier are additional components. The authors did not evaluate the approach in terms of results and just gave an idea of how this approach will work. The main drawback of this paper is that it did not present any evaluation or results as a proof of concept for their approach.

Rosario and Hearst [19] compared five generative graphical models and a neural network with lexical, syntactic, and semantic features. This paper examines the distinction among seven different relation types that exist between the disease and treatment entities. The accuracy achieved for three main relations is 92.6% for the cure, 38.5% for prevent, and 20% for side effect relations.

Analysis of previous approaches on relation extraction is summarized in Table 1.

## 3. Dataset

We used the standard text corpus that is obtained from [19]. This corpus/dataset contains eight possible types of relationships, between treatment and disease. This dataset was collected from MEDLINE 2001 abstracts. Relations are annotated from sentences taken from titles and abstracts.  Table 2 presents the original dataset, as published in previous research showing relationships and number of sentences. This dataset was collected from MEDLINE 2001 abstracts (The corpus detail and download available at the following link: http://biotext.berkeley.edu/dis_treat_data.html).

## 4. Proposed Framework

**Figure 1:**
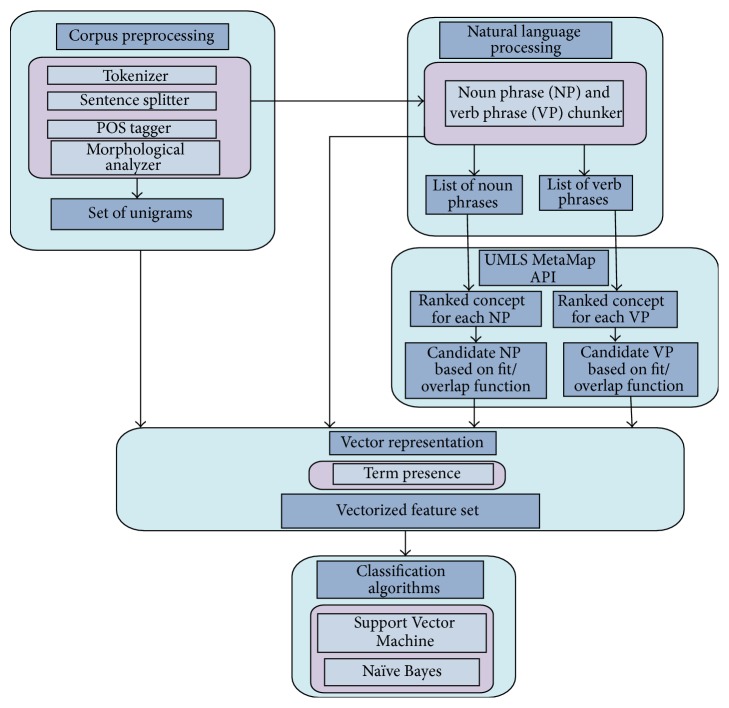
Hybrid feature set based relation extraction framework.

The framework we propose is partially inspired by Frunza and Inkpen [21], the feature set and corpus they used. However, our framework additionally uses UMLS to rank the verb phrases in the corpus, instead of only relying on noun phrase ranking. The ranking of the verb phrases using verb based biomedical lexical resource is the first implementation of this idea, to the best of our knowledge. Ben Abacha and Zweigenbaum [23] mentioned verb-related semantic resources for the medical domain, but they believe that there was no resource available at that time. Our framework for relation classification is a stepwise approach where each step deals with one module of the overall method. There are five major modules of relation extraction framework: corpus preprocessing, natural language processing, UMLS based ranking of noun and verb phrase, creation of *n*-dimensional vector space, and classification of entities.  Figure 1 shows our comprehensive, detailed proposed framework for relation extraction.

### 4.1. Corpus Preprocessing

This step is designed to preprocess the corpus by applying four text processing steps: tokenization, sentence splitting, part of speech (POS) tagging, and morphological analyzer.

General Architecture for Text Engineering (GATE) [27, 28] for text preprocessing is used, which is open source and widely used by many research communities. The purpose of the process is to transform the text that can be used for further text engineering activities. A Nearly-New Information Extraction System (ANNIE) [28] is the information extraction application available in GATE and used in our preprocessing process with default options.

The input to this step is the text corpus/dataset which is used for the task of relation classification and the output of this step is the set of unigram features that will be further used for the feature set. The preprocessing activities used in this process are

- *Tokenization*: the tokenizer splits the text into small tokens, that is, different type of words, punctuation, and numbers [29]. For example, “disease and diagnosis” has 6 tokens, that is, (disease), (space), (and), (space), (diagnoses), and (.).
- *Sentence splitting*: the sentence splitter splits the text that is required for taggers into sentences. The sentence splitter uses a dictionary list of abbreviations to differentiate between full stops and other token types [30]. Sentence splitter takes the (.) to split one sentence from another. For example, “disease and diagnosis” is a single sentence.
- *Part of speech (POS) tagging*: this module produces a part-of-speech tag and annotates each word or symbol in the text. Part of speech tags can be a verb, noun, adverb, or adjective. Tagger [31] can be customized by changing the rule set given to it. For example, “disease and diagnosis” (disease) is a noun, (and) is an article word, and (diagnosis) is also a noun.
- *Morphological Analysis*: the morphological analyzer takes as input a tokenized GATE document. It identifies the lemma and an affix of each token by considering token's part of speech tag, one at a time. These values will then be added as features on the token annotation. Morpher is based on certain regular expression rules [32]. This module is used to identify the common root of words in the text. GATE morphological analyzer is used with ANNIE to consider the root of each word, instead of the original string in feature extraction. For example, “smoking causes disease,” the word (smoking) will be converted to (smoke) and (causes) in its base form (cause).

### 4.2. Natural Language Processing

The syntactical or natural language processing (NLP) information is the second representation. It has noun phrases and verb phrases that are very important for relation classification. We consider these phrasal features with the unigrams. GENIA tagger [33] is used to extract the syntactical information from the data. GENIA tagger is specially designed for biomedical text, such as, MEDLINE abstracts. The tagger takes an English sentence as input and provides the base forms, part-of-speech tags, chunk tags, and named entity tags as outputs.

Example sentence from our dataset: “Only two protein subunits, Pop1p and Pop4p, specifically bind the RNA subunit.”

The full output of the GENIA tagger and Open NLP Chunker to extract the output of the sentence, taken as an example from our dataset, is shown in Table 3.

The noun phrases and verb phrases recognized by the tagger are considered to be the syntactical features for our technique. We preprocessed these features before finalizing the feature set, removed features that contain only punctuation, removed the stop word, and did morphological analysis to identify the root word. The purpose is to identify the base form of the word that has multiple inflected forms.

### 4.3. Noun and Verb Phrase Ranking Based on UMLS

The concept ranking module is the core contribution area of our framework, with the ranking of the verb phrases obtained in the previous phase (NLP module). The ranking uses the mapping function of MetaMap, in order to get the concept variants of noun phrases and verb phrases. Noun phrase ranking has already been used as a feature in the literature by Frunza and Inkpen [21] and got promising results. In our framework, we implemented the idea of verb phrase ranking along with noun phrases, because verb phrases are the main clue to find relation-based words in any text. The main purpose of phrasal ranking is to get domain specific concepts and to avoid noisy and irrelevant features. This domain specific phrasal ranking is the key to improve the performance of our relation extraction framework. The noun and verb phrases obtained in the NLP module of the framework are processed, in order to make them better representative feature for our relation classification.

We used UMLS [34] to rank both noun and verb phrases. This was done to measure the similarity of the concepts in the original corpus with the concept in the UMLS. MetaMap is a tool created by NLM that is used for mapping free text to medical concepts in the UMLS. UMLS consists of three knowledge sources, that is, The Metathesaurus, the Semantic Network, and the SPECIALIST Lexicon. For concept similarity or concept ranking, MetaMap uses its Metathesaurus as a knowledge source. With MetaMap API, we sent the list of noun phrases and verb phrases, which were obtained in the NLP phase of our framework. The authors in [21] also used MetaMap to rank concepts. The difference is that their ranking limits itself to noun phrases, while our method maps both noun phrases and verb phrases. Our rationale behind the use of verb phrases is very obvious, as verbs are the first indication of relation into the text. For each noun and verb phrase, variant noun and verb phrases are generated through MetaMap API. The variants or inflected forms of noun and verb phrases are then evaluated. This evaluation is done using the algorithm, which takes the list of variants generated by the MetaMap mapping function, processes them, and provides the best-ranked variant as output. The single top-ranked concept with the maximum score generated by our ranking algorithm is finally selected as a feature for our classifier. The pseudocode of our ranking algorithm is given below.

#### 4.3.1. Concept Rank Algorithm

Our algorithm consists of three major steps as follows.

**Step 1 .**

Extract related concepts for all the noun and verb phrases from UMLS.

**Step 2 .**

Filter the extracted concept for two reasons: firstly, to address the diversity of corpus, and secondly, to filter out phrases that were not related to classes on the basis of three criteria:

(1) the score of each concept which is given by MetaMap. A threshold of the concept Meta Mapping score is set as follows:
  (a) for noun phrase concepts threshold score ≥600,
  (b) for verb phrase concept threshold score ≥700,
(2) the type of each concept, that is, Therapeutic or Preventive Procedure, Functional Concept, Qualitative Concept, and so forth,
(3) if *q* − 1 dimensions of the phrase are mapped to a MetaMap concept, *q* − 1 dimensions of a phrase must exist in the MetaMap space.

**Step 3 .**

All the concepts filtered in Step 2 were selected as features for our classification algorithms.

For example the noun phrase “efficacy and safety” is extracted by the algorithm in Step 1.

The three filters in Step 2 on “efficacy and safety” will be processed as given below.

The MetaMap score of both efficacy and safety is 1000, which qualifies the 1st filter: score ≥ 600 for noun phrases.

Types of the concepts selected were [Qualitative Concept] for efficacy and [Human-caused Phenomenon or Process] for safety, and it passed the 2nd filter in Step 2.

In the 3rd filter, the phrase (efficacy and safety) has 3 words/dimensions. The words (efficacy) and (safety) match, while the word (and) does not match the MetaMap: here *q* = 3, so *q* − 1 = 2 dimensions are matched. Hence, this phrase qualifies the *q* − 1 filter.

Step 3 of the algorithm will choose “efficacy and safety” as a feature for classification.

The top two noun phrases and verb phrase obtained from noun and verb phrase chunker further processed by MetaMap API are shown in Table 4.

### 4.4. Vector Representation (Vector Space Model)

The vector space model is a representation of the documents and concepts as vectors, in a multidimensional space. Its dimensions are the terms in the documents that are used to build an index. If a term occurs infrequently in the whole collection, but frequently in the document, a high-ranking score can be assigned to that document. This phase mainly focuses on conversion of text into vectors, so it can be later used for classification. Vector is represented with the use of features, and one important decision at this stage is to select each feature weight. Feature weight also affects the classification performance as shown from literature. Bag of word (BOW) model mostly uses three feature value representations:

1. term presence or binary feature values: if the feature is present in an instance the value will be 1 and 0 otherwise;
2. frequency feature values: the value is the number of times a feature appears in an instance or 0 if it did not appear;
3. Term Frequency-Inverse Document Frequency (TFIDF): it is a relative weight of the feature with its document frequency and requires more calculation to form a vector.

We used the term presence, because it is easy to formulate a document or text vector. It requires less processing and computation in machine learning tasks and better classification results [35]. We used the term presence using the formula:

(1) $$\begin{array}{c} {\text{Feature}}_{i}=\left\{\begin{array}{cc} 1, & \text{if }\left(\text{present in document}\right),\\ 0, & \text{else.} \end{array}\right. \end{array}$$

### 4.5. Relation Classification

This phase mainly focuses on the classification of all those relations which exist between disease and treatment entities in the text corpora. Our main focus is to extract three main relations, that is, cure, prevent, and side effect relations. Support Vector Machine and Naïve Bayes algorithms are used for the classification of relations.

#### 4.5.1. Support Vector Machine Algorithm

Support Vector Machines (SVMs) belong to the group of supervised learning used for the data analysis and pattern recognition. They offer a direct and open engineering solution for classification problems. Support Vector Machines (SVMs) have also been widely used in the protein-protein interaction extraction task and have shown competitive results over other learning methods ([36, 37]). Relation extraction is a text classification problem. We used Support Vector Machine (SVM), as SVMs have already been used to yield higher accuracy on related tasks like text categorization [38]. The original implementation of SVM was designed for binary classification, while relation extraction can be a binary as well as multiclass classification problem. We have to extend the SVM for multiclass classification, for which a Library for Support Vector Machines (LIBSVM) [39] is used that is integrated software for support vector classification. We used the LIBSVM in both settings, that is, linear kernel and Radial Based Function (RBF) kernel. For linear kernel, the best results are obtained at *c* = 0.5, while other parameters are on default settings. For RBF kernel, best results are when *g* = 0.05 and *c* = 8.

When an SVM is used for classification, it is important that an appropriate kernel function is chosen. For classification tasks such as relation extraction, where the number of feature set is large, it has been reported [40] that a linear kernel is typically the most suitable. The authors of paper [41] also compared three types of SVM kernels (linear, quadratic, and cubic kernels) for relation extraction task. In their comparison, they reported that, with all the features, the linear kernel is better than both the quadratic and cubic ones.

The dataset used in our research consists of high-dimensional feature space. It has been reported [42] that SVMs build a separating hyper plane in a high-dimensional feature space in order to maximize the separability. This hyper plane uses the small training vectors, called the support vectors, in the original space. For a given finite set of learning patterns, the optimum separation hyper plane is the linear classifier with a maximum margin between the positive and the negative samples. The problem of relation extraction normally is not a binary classification task. So, before using the SVMs, it is mandatory to first reduce the relation extraction to a binary or multiclass classification process.

#### 4.5.2. Naïve Bayes Algorithm

It is a probabilistic classifier which applies Bayes' theorem with strong independence assumptions on features and the order is irrelevant. Thus, the presence of one feature does not affect other features in classification tasks. Due to the precise nature of the probability model, the Naïve Bayes classifiers can be efficiently trained by the comparatively less amount of data to estimate the classification parameters. Due to the independence of variables, for each class, only the variances of the variables need to be determined, not the entire covariance matrix. We use the Naïve Bayes algorithm in our experimentation because the prevent and side effect relations have a very small amount of training data that is the main advantage of the Naïve Bayes classifier. The simplicity of Naïve Bayes also makes it attractive in numerous tasks with reasonable performance. Recently, [43] reported significant results for text classification by Naïve Bayes with SVM. Below are the general equations of the Naïve Bayes classifier:

(2) Pci ∣ D=PciPD ∣ ciPD,PD ∣ ci=∏j=1nPdj ∣ ci.

Naive Bayes performs well on numeric and textual data, it is easy to implement and computationally simple as compared to other algorithms. However, due to conditional independence assumption, its performance is affected when data has correlated features.

## 5. Experimental Design

The settings are also taken from [21]; the reason for reusing these settings was ensuring the authenticity and comparison of results.

*Setting 1*. In this setting, we set up three models and two classes labeled as positive and negative. In the first model, positive class is cure, while negative class is Disonly, Treatonly. For the second model positive class is prevent relation, negative class once again is Disonly, Treatonly. For the third model, side effect is positive and Disonly, Treatonly are negative. 

*Setting 2*. Here, we built three models again, each focused on one relation that can distinguish sentences that contain the relation, from sentences that do not contain any relevant information. The first model has cure as positive class and vague as negative class. In the second model, prevent relation is positive, while vague is negative whereas, in the third model, side effect is positive while vague is negative again. 

*Setting 3*. Once again three models were built that distinguish the three relations: we have cure as positive, and we have prevent and side effect relations as negative. Prevent relation is positive and side effect is negative and side effect is positive and prevent relation is taken as negative class. This is a special setting in which all the three meaningful relation sentences (cure, prevent, and side effect) are considered as positive and negative alternatively, in order to check the performance of our feature set, so that the resultant relation is chosen accurately.

The detail of each setting is explained as in Table 5.

## 6. Results and Discussion

Table 6 presents the results of our framework on the dataset for cure, prevent, and side effect relations. We chose these three relations from the dataset because these are the only meaningful relation types that exist in the dataset. Our dataset is designed by [19] and already used in research [21, 23] with different settings for cure, prevent, and side effect relations. Ben Abacha and Zweigenbaum [23] used three different settings for each type of relation; the same settings are being used in our case. Our framework mainly focused on four types of features that have been extracted from the sentences in the datasets. This feature set consists of bag of word features (unigram), natural language processing features (verb and noun phrases), filtration of noun phrases features using UMLS, and filtration of verb phrases features from the UMLS.

We also used SVM and Naïve Bayes algorithms to classify our feature set because of the following reasons: SVM is used by many researchers as a baseline classifier for text classification task, and it is the most widely used classifier for text classification [43]. Naïve Bayes is used particularly for those example sets in data that have very less participation in the overall dataset. In our case, prevent and side effect relations have only 63 and 30 sentences available. Both of these relations showed exceptional results for Naïve Bayes, as it requires small training data to learn the classifier. We swapped the role of data; that is, the data used for training will be used for testing, and the data used for testing will be used for training. We used 10-fold cross validation when there is a limited amount of data for training and testing. 10-fold means, we split the data into 10 equal partitions and the process for 10 times, while ensuring that each partition is used for testing at once, such that 10% data is for testing and 90% is for training. Average the performance results of 10x iterations to get the final results. Table 6 shows the results of our framework in detail.

Table 6 is showing the results of all three settings with the combination of different feature sets. Accuracy, precision, recall, and *F*-score are the metrics used to evaluate the performance of our approach.

For cure relation, our approach achieved the best *F*-scores in settings 1, 2, and 3 as 85.10% (SVM linear) with BOW representation, 98.05% (SVM-RBF kernel) with our proposed UMLS (NP + VP) feature set, and 97.58% (SVM-RBF kernel) with UMLS (NP), respectively. It can be observed that, for cure relation, the overall best results of *F*-score are achieved with our proposed feature set.

For prevent relation, we achieved best *F*-scores in settings 1, 2, and 3 as 81.42% (SVM linear) with UMLS (NP), 93.55% (NB) with our proposed UMLS (NP + VP) feature set, and 91.06% (NB) with BOW representation, respectively. Again in this case the overall best *F*-score for prevent relation is achieved with our proposed feature set.

For side effect relation, the achieved best *F*-scores in settings 1, 2, and 3 as 30.43% (NB) with NLP, 74.20% (NB) with BOW representation, and 88.89% (NB) with our proposed UMLS (NP + VP), respectively. Once again, it can be observed that for side effect relation the overall best results of *F*-score are achieved with our proposed feature set. All the results for precision, recall metrics can be analyzed from Table 6.

Table 7 presents a comparison of the accuracy results obtained in the previous work by [19, 21] and our proposed approach. As we can see from the table, our technique has a major edge over previous results for all three relations. Our results are very consistent in terms of accuracy for all three relations. The improvement for cure relation is 1.19 percent points; prevent improved by 22.45 percent points, and side effect improved by 50.49 percent points in terms of accuracy.

Table 8 compares our *F*-measure results with state-of-the-art approaches applied on the same corpus. Our approach clearly outperforms the work presented by [23] and the work of [21]. Frunza and Inkpen [21] reported good results on the same corpus in setting 3. However, these results are not reproducible using 10-fold cross validation. We used the same settings and got those results by training the classifier on 90% data and testing it on 10%. This shows that the results quoted in [21] are looking biased due to training and testing ratio.

Our results mainly improved due to the reason that we used verb-based concept ranking from UMLS. Verb phrases are the main features for the relations in the text. All other features used in this approach are already used for text classification, except the ranking of verb phrases using UMLS. We ranked both the noun and verb phrase concepts using an algorithm in which we used filters to rank all the concepts on the basis of three different criteria. This improved our results significantly compared to the previous approaches on the same dataset. Our results are effective due to 10-fold cross validation and efficient in terms of *f*-measure and accuracy.

## 7. Conclusion and Future Directions

This research was aimed to build a relation extraction framework between medical entities from biomedical texts. We mainly focused on the extraction of semantic relations between treatments and diseases. The proposed approach relies on a hybrid feature set, which consists of (1) bag of word model, (2) natural language processing features, (3) lexical features, and (4) semantic features based on UMLS concepts. We used the supervised learning methods that used SVM and NB classifier to evaluate our feature set. We conducted experiments on this approach and compared it with the previous approaches [19, 21, 23]. The obtained results showed that our approach clearly outperformed the previous techniques and provided an alternative to improve the accuracy and *F*-measure of relation extraction in the biomedical domain: few training examples are available.

In future, we intend to test our approach with other types of relations and different corpora; we will also work on multistage classifier to enhance the performance of relation extraction. Some unsupervised techniques can be introduced for relation classification.

## Figures and Tables

**Table 1:** Analysis of the existing literature on biomedical relation extraction.

Authors	Technique	Domain area	Type of relations	Year of publication	Reported results
Huang et al. [20]	Hybrid approach (shallow parsing and pattern matching)	Biomedical	Protein-protein (P2P) interaction	2006	80% *F*-score

Frunza and Inkpen [21]	Hybrid approach	Biomedical	Disease and treatment relation (cure, prevent, and side effect relations)	2010	Accuracy Cure 95% Prevent relation 75% Side effect 46%

Sharma et al. [22]	Verb-centric algorithm	Biomedical	Not mentioned	2010	90% *F*-score

Ben Abacha and Zweigenbaum [23]	Hybrid approach (pattern based and machine learning)	Biomedical	Disease and treatment relation	2011	94.07% *F*-score

Ben Abacha and Zweigenbaum [24]	Linguistic patterns and domain knowledge	Biomedical	Relation between 16 entities	2011	Precision of 75.72% and recall of 60.64%

Yang et al. [25]	Verb-centric approach	Biomedical	Relation between (foods, chemicals, diseases, proteins, and genes)	2011	90.5% *F*-score

Kadir and Bokharaeian [26]	Hybrid approach (rule-based, kernel based, and cooccurrence based methods)	Biomedical	Not mentioned	2013	Not reported

Rosario and Hearst [19]	Graphical models and neural network	Biomedical	Disease and treatment relation (cure, prevent, side effect relations)	2004	Accuracy Cure 92.6% Prevent relation 38.5% Side effect 20%

**Table 2:** Original dataset description from [21].

Sr. number	Relationship	Number of sentences
1	Cure/treat for dis.	810
2	Prevent relation	63
3	Side effect	29
4	DisOnly	616
5	TreatOnly	166
6	Vague	36
7	No cure/treat number for dis.	4
8	Nonrelevant	1771
Total		3495

**Table 3:** GENIA tagger output on the example sentence.

Word	Base form	POS	Chunk	Named entity	Open NLP
Only	Only	RB	B-NP	O	B-NP
Two	Two	CD	I-NP	O	I-NP
Protein	Protein	NN	I-NP	B-protein	I-NP
Subunits	Subunit	NNS	I-NP	I-protein	I-NP
Pop1p	Pop1p	NN	B-NP	O	B-NP
And	And	CC	I-NP	O	I-NP
Pop4p	Pop4p	NN	I-NP	O	I-NP
Specifically	Specifically	RB	B-ADVP	O	B-ADVP
Bind	Bind	VBP	B-VP	O	B-VP
The	The	DT	B-NP	O	B-NP
RNA	RNA	NN	I-NP	B-protein	I-NP
Subunit	Subunit	NN	I-NP	I-protein	I-NP

**Table 4:** Output of MetaMap API to rank the noun phrases and verb phrases.

S/number	VP	UMLS concept	NP	UMLS concepts
1	Experience cover place too	Meta mapping (775): 812 experiences [Mental Process] 812 experiences (practical experience) [Mental Process] 812 covers (cover, action) [Functional Concept] 812 covers (covers) [Medical Device] 812 covers (cover device component) [Medical Device] 812 places [spatial concept] 812 places (place, dosing instruction imperative) [Functional Concept] 812 places (put, instruction imperative) [Activity]	The abdomen diagnostic peritoneal lavage 4 g/p and amp	Meta mapping (599): 626 abdomens [Body Location or Region] 626 abdomens (abdominal cavity) [Body Location or Region] 626 abdomens (entire abdomen) [body part, organ, or organ component] 668 diagnostic peritoneal lavages (peritoneal lavage) [diagnostic procedure, Therapeutic or Preventive Procedure] 626 g% (gram per deciliter) [Quantitative Concept]

2	be to compare	Meta mapping (1000): 1000 to [Qualitative Concept] 1000 to (Togo) [Geographic Area] 1000 to (tryptophanase) [amino acid, peptide, or protein, and enzyme] 1000 comparisons (comparison) [Activity]	Efficacy and safety	Meta mapping (1000): 1000 efficacy concepts (effectiveness) [Qualitative Concept] 1000 efficacy concepts (Efficacy Study) [Research Activity] 1000 safety concepts [Human-caused Phenomenon or Process] 1000 safety concepts (safety study) [Research Activity]

**Table 5:** Detail of experimental settings.

Setups	Class label: +1	Class label: −1
Setting # 1	Cure	Disonly + Treatonly
Setting # 1	Prevent	Disonly + Treatonly
Setting # 1	Side effect	Disonly + Treatonly
Setting # 2	Cure	Vague
Setting # 2	Prevent	Vague
Setting # 2	Side effect	Vague
Setting # 3	Cure	Prevent + side effect
Setting # 3	Prevent	Side effect
Setting # 3	Side effect	Prevent

**Table 6:** Results of all feature sets using classification algorithms.

Relation	Feature set	Classification performance of all feature sets											
		Setting 1				Setting 2				Setting 3			
		Algo	FS	P	R	Algo	FS	P	R	Algo	FS	P	R
Cure	BOW^*∗*^	SVM-RBF	84.76	85.07	84.46	SVM-RBF	97.87	96.38	99.4	SVM-RBF	97.58	95.82	99.4
		SVM	**85.1**	86.64	83.61	SVM	97.99	96.28	99.76	SVM	97.45	96.13	98.8
		NB	82.88	84.48	81.33	NB	97.26	96.34	98.19	NB	96.45	94.77	98.19
	BOW + NLP^*∗∗*^	SVM-RBF	84.61	86.14	83.13	SVM-RBF	97.92	96.38	99.52	SVM-RBF	97.29	95.37	99.28
		SVM	84.53	85.84	83.25	SVM	97.74	96.59	98.92	SVM	97.03	95.67	98.43
		NB	83.64	83.8	83.49	NB	97.19	96.44	97.95	NB	96.51	94.88	98.19
	BOW + NLP + UMLS (NP)^*∗∗∗*^	SVM-RBF	84.9	85.47	84.34	SVM-RBF	97.99	96.39	99.64	SVM-RBF	**97.58**	95.82	99.4
		SVM	84.44	86.58	82.41	SVM	97.98	96.6	99.4	SVM	97.08	95.89	98.31
		NB	82.71	84.81	80.72	NB	97.06	96.42	97.71	NB	96.45	94.66	98.31
	BOW + NLP + UMLS (NP + VP)^*∗∗∗∗*^	SVM-RBF	84.83	85.82	83.86	SVM-RBF	**98.05**	96.39	99.76	SVM-RBF	97.41	95.49	99.4
		SVM	84.83	86.87	82.89	SVM	97.92	96.6	99.28	SVM	97.08	95.89	98.31
		NB	82.71	84.53	80.96	NB	96.94	96.42	97.47	NB	96.58	94.68	98.55

Prevent relation	BOW^*∗*^	SVM-RBF	78.95	88.24	71.43	SVM-RBF	90.91	94.83	87.3	SVM-RBF	89.06	87.69	90.48
		SVM	81.02	93.75	71.34	SVM	91.81	94.92	88.89	SVM	86.82	84.85	88.89
		NB	60.2	77.5	49.21	NB	91.94	93.44	90.48	NB	**91.06**	93.33	88.89
	BOW + NLP^*∗∗*^	SVM-RBF	71.15	90.24	58.73	SVM-RBF	90.75	96.43	85.71	SVM-RBF	86.61	85.94	87.3
		SVM	80	93.62	69.84	SVM	90.91	94.83	87.3	SVM	86.15	83.58	88.89
		NB	63.07	72.92	55.56	NB	93.55	95.08	92.06	NB	88.71	90.16	87.3
	BOW + NLP + UMLS (NP)^*∗∗∗*^	SVM-RBF	72.38	90.48	60.32	SVM-RBF	90.75	96.43	85.71	SVM-RBF	88.19	87.5	88.89
		SVM	**81.42**	92	73.02	SVM	90.91	94.83	87.3	SVM	86.15	83.58	88.89
		NB	62.13	80	50.79	NB	93.55	95.08	92.06	NB	88.72	90.19	87.3
	BOW + NLP + UMLS (NP + VP)^*∗∗∗∗*^	SVM-RBF	72.38	90.48	60.32	SVM-RBF	90.75	96.43	85.71	SVM-RBF	85.94	84.62	87.3
		SVM	80.36	91.84	71.43	SVM	90.91	94.83	87.3	SVM	85.5	82.35	88.89
		NB	62.26	76.74	52.38	NB	**93.55**	95.08	92.06	NB	88.89	88.89	88.89

Side effect	BOW^*∗*^	SVM-RBF	21.05	50	13.33	SVM-RBF	70	70	70	SVM-RBF	75.86	78.57	73.33
		SVM	21.74	31.25	16.67	SVM	63.16	66.67	60	SVM	70.18	74.07	66.67
		NB	22.22	33.33	16.67	NB	**74.2**	71.88	76.67	NB	83.54	78.79	88.89
	BOW + NLP^*∗∗*^	SVM-RBF	25.65	55.56	16.67	SVM-RBF	68.97	71.43	66.67	SVM-RBF	71.18	72.41	70
		SVM	22.73	35.71	16.67	SVM	65.52	67.86	63.33	SVM	67.86	73.08	63.33
		NB	**30.43**	43.75	23.33	NB	67.69	62.86	73.33	NB	77.42	75	80
	BOW + NLP + UMLS (NP)^*∗∗∗*^	SVM-RBF	16.67	50	10	SVM-RBF	66.67	66.67	66.67	SVM-RBF	74.57	75.86	73.33
		SVM	18.18	28.57	13.33	SVM	62.07	64.29	60	SVM	67.86	73.08	63.33
		NB	13.64	21.43	10	NB	68.75	64.71	73.33	NB	88.71	90.16	87.3
	BOW + NLP + UMLS (NP + VP)^*∗∗∗∗*^	SVM-RBF	21.62	57.14	13.33	SVM-RBF	70.18	74.07	66.67	SVM-RBF	68.97	71.43	66.67
		SVM	22.22	33.33	16.67	SVM	65.52	67.86	63.33	SVM	65.45	72	60
		NB	26.67	40	20	NB	67.69	62.86	73.33	NB	**88.89**	88.89	88.89

^*∗*^Bag of word (BOW) is unigrams only.

^*∗∗*^Natural language processing (NLP) is noun and verb phrases.

^*∗∗∗*^UMLS (NP) is the use of UMLS with noun phrase ranking only.

^*∗∗∗∗*^UMLS (NP + VP) is the use of UMLS with noun phrase and verb phrase ranking.

SVM is Support Vector Machine algorithms; SVM-RBF is Support Vector Machine-Radial Based Function algorithm; NB is Naïve Bayes algorithm; FS is *F*-score; P is precision; R is recall.

**Table 7:** Classification of accuracy comparison with state-of-the-art approaches.

Relations	Comparison of accuracy results		
	Rosario and Hearst [19]	Frunza and Inkpen [21]	Our approach
Cure	92.6%	95%	96.19%
Prevent relation	38.5%	75%	97.45%
Side effect	20%	46%	96.49%

**Table 8:** *F*-measure comparison with state-of-the-art approaches.

Relations	Comparison of *F*-measure results		
	Frunza and Inkpen [21]	Ben Abacha and Zweigenbaum [23]	Our approach
Cure	87.10	96.84	98.05
Prevent relation	77.78	67.92	93.55
Side effect	55.56	64.15	88.89
